# Applications and Implications of Heparin and Protamine in Tissue Engineering and Regenerative Medicine

**DOI:** 10.1155/2014/936196

**Published:** 2014-06-03

**Authors:** Judee Grace E. Nemeno, Soojung Lee, Wojong Yang, Kyung Mi Lee, Jeong Ik Lee

**Affiliations:** Regenerative Medicine Laboratory, Center for Stem Cell Research, Department of Biomedical Science and Technology, Institute of Biomedical Science & Technology (IBST), Konkuk University, Seoul 143-701, Republic of Korea

## Abstract

Drug repositioning is one of the most rapidly emerging fields of study. This concept is anchored on the principle that diseases have similar damaged or affected signaling pathways. Recently, drugs have been repositioned not only for their alternative therapeutic uses but also for their applications as biomaterials in various fields. However, medical drugs as biomaterials are rarely focused on in reviews. Fragmin and protamine have been recently the sources of increasing attention in the field of tissue engineering and regenerative medicine. Fragmin and protamine have been manufactured primarily as a safe antidote for the circulating heparin. Lately, these drugs have been utilized as either micro- or nanoparticle biomaterials. In this paper, we will briefly describe the concept of drug repositioning and some of the medical drugs that have been repurposed for their alternative therapeutic uses. Also, this will feature the historical background of the studies focused on fragmin/protamine micro/nanoparticles (F/P M/NPs) and their applications as biomaterials in tissue engineering, stem cell therapy, and regenerative medicine.

## 1. Introduction

Since the birth of tissue engineering, various diseases have been addressed through the biofabrication of engineered cells or tissues, either made up of scaffolds or scaffold-free. In congruence to this, drug repositioning strategy has also progressed and has been significantly applied in various therapeutic strategies including drug-based and stem cell-based therapies through their applications in generating biomimetic constructs. Hence, advancement in tissue engineering as well as regenerative medicine has been enhanced due to some repositioned drugs.

Originally, drug rescue or repositioning is deemed as an effective strategy against the laborious and highly expensive* de novo* drug discovery as well as stagnant approval system of new drugs [1]. The first repositioned drug was actually an abandoned medicine, zidovudine (azidothymidine), for cancer due to lack of evidence for its effectiveness. However, it was later discovered to be effective in treating patients who are suffering from human immune deficiency virus (HIV) or acquired immunodeficiency syndrome (AIDS) [2, 3]. From then on, many groups have been motivated to explore other approved drugs for their alternative therapeutic uses.

Lately, fragmin and protamine have been few of the leading drugs repurposed as biomaterials in tissue engineering, stem cell therapy, and regenerative medicine. Fragmin and protamine have been utilized as biomaterials, either as microparticles (MPs) or as nanoparticles (NPs), in designing tissue constructs. Recent studies show that they are useful in cell-based therapy in various fields including cardiovascular [4, 5], dental [6], and dermatological medicine [7, 8].

In this review, we will briefly describe the concept of drug repositioning and some of the drugs that have been repurposed for other indications. Also, this will feature the historical background of the studies focused on fragmin/protamine micro/nanoparticles (F/P M/NPs) and their applications as biomaterials in tissue engineering, stem cell therapy, and regenerative medicine.

## 2. Review

### 2.1. Drug Repositioning Strategy and Its Implications in Tissue Engineering and Regenerative Medicine

Drug repurposing or repositioning strategy has been rapidly emerging in the recent years [2, 9]. The trend shows that there is an increasing pattern of drug repositioning research in the academic field rather than the industrial area parallel to the shift of the greater number of scientists in the academe from the pharmaceutical industries [10]. Thus, it is expected that a number of drug discoveries and drug repositioning effort will continue to accumulate from these academic institutions compared to those that can be generated from the pharmaceutical companies.

Initially, this strategy was adapted to address the pitfalls of the previous research and development related to the interactions, safety, and efficacy of drugs. Repurposing drugs involves the reevaluation of the currently approved or even the abandoned drugs for their alternative therapeutic potentials [2]. This strategy has efficiently reduced the high cost of traditional and time-consuming drug testing and screening, lessened the clinical risks, and accelerated the progress of academic research focused not only on approved drugs but also on safety-tested but unapproved drugs [10, 11].

Since the repositioning of AZT [3] in 1987, a number of drugs have been repurposed for their alternative indications. To name a few, Viagra (Sildenafil) [12], a drug for angina pectoris and hypertension, has been used to treat erectile dysfunction; aspirin which was originally manufactured as an antipyretic and an anti-inflammatory drug has been indicated for hypertension and antiplatelet aggregation and has been established to exhibit cardiac protective function [13]; Rogaine (Minoxidil), an antihypertensive drug, has been used to treat baldness [14]; and tocilizumab, an antirheumatism drug, has been lately demonstrated to have therapeutic effects on amyotrophic lateral sclerosis (ALS) [15] and cancer [16]. The rest of the repositioned drugs during the 20th century include Amphocin (Amphotericin B), Rogaine (Minoxidil), Thalidomide (Thalidomide), Avodart (Dutasteride), Neurontin (Gabapentin), Symmetrel (Amantadine), Zyban (Bupropion), ReQUIP (Ropinirole), Aspirin (acetylsalicylic acid), Gemzar (Gemcitabine), Evista (Raloxifene), Viagra (Sildenafil), Yentreve (Duloxetine), Trexall (Methotrexate), and Parlodel (Bromocriptine) [17–28]. A summary of these drugs, along with their alternative therapeutic uses and their respective year of repurposing, is presented in Table 1.

Lately, a number of drugs have been applied in tissue engineering and regenerative medicine including warfarin [29], avastin [30–32], and a number of parenteral drugs [33]. Moreover, fragmin and protamine, either as MPs or as NPs, have been the leading drugs repurposed as biomaterials in tissue engineering and regenerative medicine.

### 2.2. Historical Background of Fragmin/Protamine Micro/Nanoparticles as a Drug Repositioning Strategy

Fragmin and protamine have been progressively used not only in the field of tissue engineering but also in regenerative medicine. The use of protamine under the concept of drug repositioning dates back to the 1930s when this positively charged polypeptide, obtained from the sperm of California rainbow trout, was added to the neutral protamine hagedorn insulin to prolong its effect in maintaining hypoglycemia up to 24 hours in patients who have severe diabetes mellitus [34, 35]. The long-lasting effect of insulin-protamine combination was further enhanced when a certain amount of zinc was used to stabilize the complex and it was proven to sustain low blood glucose level up to three days after injection in diabetic patients [36]. In the later decades, protamine has been utilized primarily as a safe antidote for the circulating heparin after stent implantation. Administration of protamine demonstrated beneficial effects since it reduced the hospital stay of the patients free of medical complication after stent implantation [37]. The succeeding report showed that heparin has been clinically utilized as the prophylaxis and treatment of deep venous thrombosis (DVT) and that their molecular weight distributions significantly influence their binding properties with protamine [38].

Later on, these F/P MPs have been utilized as carriers of various stem cells, namely, adipose tissue-derived stromal cells (ASCs) and inbred rat adipose tissue-derived stromal cells (IR-ASCs) in an ordinary monolayer and a 3D culture system, respectively. The data revealed that F/P MPs improved the viabilities of the cells, induced their eventual aggregation, increased the proliferation rate, and promoted speedy neovascularization and tissue granulation after transplantation [39–41]. Moreover, F/P MPs have been found to be efficient cell carriers not only for single cell types but also for cocultured cells such as that in the 3D expansion of ASCs and bone marrow-derived mesenchymal stem cells (BMSCs). The cocultured cells also exhibited rapid proliferation rate and multilineage differentiation while maintaining their markers in spite of low serum content during the culture period [42]. Recently, F/P MPs coating method is useful for maintaining the quiescent state of rat hepatic stellate RI-T cells (HSCs) with low expressions of collagen I*α*I and TGF-*β* 1 mRNA levels [43].

Furthermore, another report demonstrated that F/P MPs can function as immobilizers or attractants of various cytokines as well as deliberate controllers for drug release. F/P controlled the gradual release of heparin-binding growth factors like fibroblast growth factor-2 (FGF-2) and cytokines such as interleukin- (IL-) 3, granulocyte-macrophage colony-stimulating factor (GM-CSF), stem cell factor (SCF), thrombopoietin (Tpo), and Flt-3 ligand into the culture media. Consequently, F/P promoted the superior cell growth of human microvascular endothelial cells (hMVECs), human dermal fibroblast cells (hDFCs), hematopoietic cell line (TF-1), hematopoietic progenitor cells (HCs), human ASCs, and BMSCs. Therefore, it is notable that F/P M/NPs serve as coating biomaterials that adequately regulate the heparin-binding cytokines responsible for controlling cellular growth and differentiation [41, 42, 44].

It has also been found that F/P M/NPs can control the release of FGF-2 and significantly promote neovascularization and tissue formation one week after they were coinjected at the injured site. A recent study was conducted using mice models where the mitogenic effects of hepatocyte growth factor- (HGF-) containing F/P MPs on cultured MVECs and their angiogenic potentials were evaluated. The results of the study suggest that HGF is highly activated, parallel to the adsorption of F/P MPs, and that HGF release is correlated to the diffusion and/or biodegradation of the F/P MPs. Finally, HGF-containing F/P MPs induce substantial cell proliferation and vascularization* in vivo*. This increased angiogenic activity of HGF* in vivo* was probably due to both sustained local release and protection against biodegradation by the F/P MPs [5]. Also, F/P MPs promoted collateral vessel formation in peripheral artery disease (PAD) models when applied with FGF-2 [4, 45].

Another important use of F/P MPs is that they serve as competent carriers of the proteins present in human platelet-rich plasma (PRP) that stimulate neovascularization and granulation tissue formation. F/P MPs effectively adsorb growth factors and thereby confirmed the previous finding that F/P MPs can significantly enhance neovascularization and filtration of inflammatory cells [41, 46]. Alternatively, the preinjection of PRP and F/P MPs also represents a promising treatment to prevent skin flap necrosis in reconstructive surgery. The study demonstrated that when PRP and F/P MPs were injected into the damaged skin prior to elevation, a notable cell survival and reduction of necrosis were observed [8].

Lately, protamine has been found to be a suitable injectable biomaterial in the field of dental medicine. A DNA/protamine complex paste with appropriate viscosity has demonstrated a bacteriostatic rather than bacteriocidal effect as it efficiently inhibited growth of bacterial species when injected as a dental implant, a drug carrier for gum pocket treatment, and also promoted guided tissue regeneration (GTR) and guided bone regeneration (GBR). Few of the oral bacterial species that can be controlled by the DNA/protamine complex paste include* Staphylococcus aureus, Pseudomonas aeruginosa, Porphyromonas gingivalis, and Prevotella intermedia *[6].

Furthermore, dalteparin (F; identical to fragmin) and protamine microparticles (F/P MPs) showed a promising therapeutic use in the field of dermatology particularly on hair reconstruction in order to address alopecia. Combined application of PRP and F/P MPs as well as PRP alone facilitated hair growth but F/P MPs provided additional hair growth. It has been demonstrated that PRP and F/P MPs induced increase in epithelial cells, collagen synthesis, and fibroblasts proliferation that facilitated rapid hair growth [7]. This advancement on hair regeneration will offer benefit to a number of patients suffering from the different forms of alopecia, a hair loss medical problem. A summary of the uses of fragmin/protamine under the concept of drug repositioning in tissue engineering and regenerative medicine is presented in Table 2.

**Figure 1:**
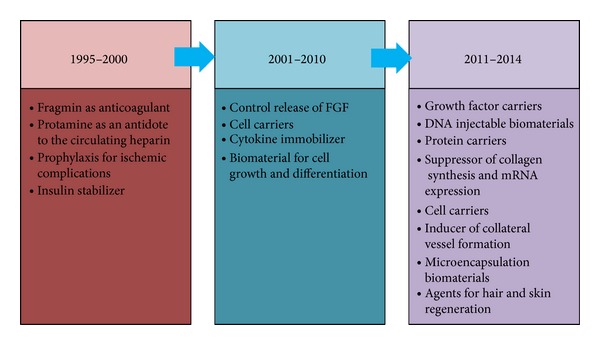
Evolution of fragmin and protamine in tissue engineering and regenerative medicine under the concept of drug repositioning. In the mid-1990s, F/P have been primarily used as antagonists of the circulating heparin. By the year of 2000, these drugs have been repurposed as biomaterials that control growth factor release, cell carriers, and cytokine attractant or immobilizer. Lately, more repositioning strategies have been emerging including DNA injectable complex, collagen and mRNA suppressor, protein carriers, and microencapsulation agents.

Taken together, the uses of fragmin and protamine have evolved from being simple drugs against thromboembolism to DVT prophylaxis, and eventually as insulin stabilizer in the mid-1990s. In the succeeding decade, these drugs have been repurposed as carriers of various types of growth factors, cells, and cytokines while influencing cell growth and differentiation. Additional uses of F/P MPs have been discovered in the recent times as a DNA carrier dental biomaterial, protein carrier, suppressor of collagen, and mRNA expression and an inducer of neovascularization (Figure 1).

### 2.3. Recent Advancement of the Use of Fragmin/Protamine Nanoparticles in Tissue Engineering and Regenerative Medicine

As the research on stem cell therapy continues to rise, search for the alternative uses of fragmin/protamine also increases dramatically. Hence, our group has also conducted such studies with the goal of producing significant contribution to the field of tissue engineering and regenerative medicine. The succeeding sections include the details of our recent findings. Although most of the studies featured in this paper were focused on F/P MPs, our recent work has been focused on F/P NPs applied as encapsulation and cell aggregation biomaterials.

#### 2.3.1. Use of Nanoparticles for Microencapsulation of Isolated Islets

Due to the increasing morbidity and even mortality brought about by diabetes mellitus, a number of studies have been conducted focusing on engineering pancreatic islets [47–49]. We have previously reported that artificial islets can be manufactured from a single cell type, the rat insulin-secreting cell line (RIN-5F), or by coculturing two different cell lines such as RIN-5F and Hep-G2 under shaking mechanism during the culture period [50, 51]. These pseudoislets can be eventually used as bioartificial pancreas for* in vitro* functional analysis and drug testing. Further applications of F/P MPs in engineering pancreas derived from primary stem cells require further studies in order to achieve transplantable bioartificial pancreas. Although, in transplantation, immune complication remains to be a limiting factor that necessitates the recipient to take immune suppression drugs such as cyclosporine A, these immune suppression drugs induce various side effects [52]. Therefore, immunoisolation by using various artificial encapsulation materials has been developed; however, their incomplete biocompatibility causes foreign body reaction against the membranes while many other side effects remain unknown up till the present. For these reasons, the enhancing immunoisolation strategies have been the primary source of interests of the current researches. The term “immunoisolation” refers to the encapsulation of a graft in a selectively permeable membrane to purposively provide protection of the graft against autoimmune responses as well as reduce the occurrence of graft rejection without using immunosuppressive agents. We have developed chondrocyte sheets that can be utilized to encapsulate bioartificial tissues or even cells as new techniques for macroencapsulation and microencapsulation, respectively [53]. Also, we have conducted an investigation that has been focused on pancreatic islet encapsulation using chondrocytes [54]. In addition, our recent study shows that F/P NPs have excellent potentials as biomaterials since they promoted speedy cell encapsulation of the pancreatic islets within a shorter period (unpublished data).

#### 2.3.2. H/P as Cell Carriers—Cell Aggregation

We recently investigated the adhesive properties of F/P as carriers of various stem cells. Our data demonstrated that large, viscous cellular aggregates composed of MSCs and H9C2 cells can be easily manufactured at a relatively shorter period when they were mixed with F/P MPs. In this study, the cells cultured via monolayer system were trypsinized and then mixed with different cell delivery agents, including D-PBS, culture medium, fragmin alone, protamine alone, F/P MPs, and mixed lyophilized F/P MPs. In comparison to other cell delivery agents, cells mixed with F/P MPs exhibited superior cell viscosity and adhesion properties [55]. When these cellular aggregates with F/P MPs were transplanted into a cartilage defect model, the cells remain intact and prevented from spreading away from the transplanted site (unpublished data). Taken together, F/P MPs are small molecules that enhance cell aggregation which may be attributed by the opposing electrical charges of both the negatively charged fragmin and the positively charged protamine [55]. F/P MPs are potential, less-expensive alternatives of cytodex and are efficient microcarriers of cells. Therefore, cell aggregation induced by F/P MPs may improve the efficiency of cell therapy and may represent a novel method for cell transplantation.

## 3. Conclusions and Future Directions

**Figure 2:**
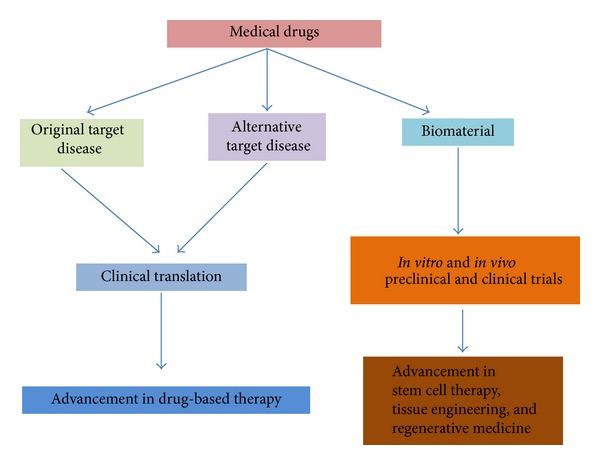
The concept of drug repositioning strategy. Drug repurposing strategy has not only brought the progress of drug-based therapy but also paved the way to the advancement of stem cell therapy, tissue engineering, and regenerative medicine.

In this paper, we have presented an overview on the rapid and profitable contributions of drug repositioning strategies to the advancement of a number of emerging fields of scientific researches. Firstly, drug-based therapy has improved when the medically approved drugs or previously abandoned drugs have been rediscovered for other indications. Secondly, parallel to the manufacturing of advanced tissue-engineered biomaterials in both the preclinical and clinical translation, a remarkable progression of the application of stem cell therapy in regenerative medicine is also notable. In particular, F/P M/NPs are just some of the drugs that lead to the robust development of stem biology, tissue engineering, and regenerative medicine (Figure 2).

**Figure 3:**
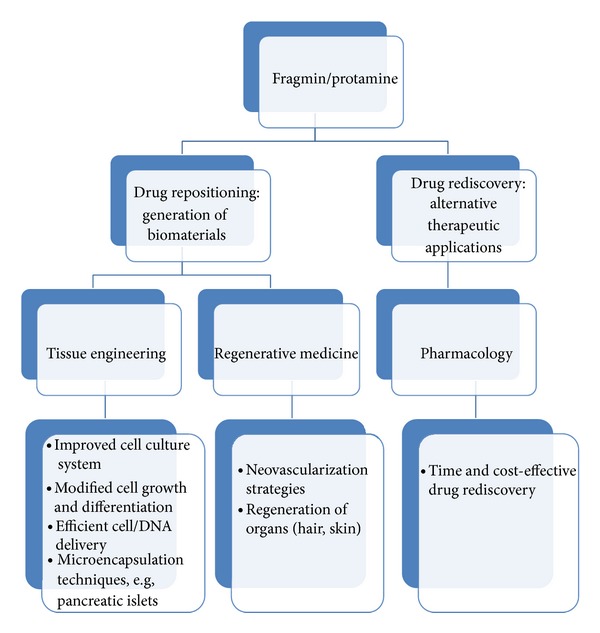
Fragmin/protamine repurposing expedited progress of pharmacology, tissue engineering, and regenerative medicine. Repositioning of F/P paved the way to a time and cost-effective drug rediscovery while their applications of F/P in tissue engineering and regenerative medicine resulted in cell culture systems; modified cell growth and differentiation; efficient protein, cell, and DNA delivery; and enhanced microencapsulation techniques, neovascularization, and regeneration of tissues/organs.

Over and beyond the time-efficient and cost-effective drug rediscovery, drug repositioning strategies had paved the way to the significant applications of these drugs as promising biomaterials in tissue engineering such that F/P has improved cell culture systems, modified cell growth and differentiation, ensured efficient cell/DNA delivery to the target damaged tissues, and possibly manufactured bioartificial pancreatic islets through microencapsulation techniques. Furthermore, F/P has been demonstrated to enhance neovascularization and rejuvenation of damaged organs, including hair and skin flaps (Figure 3). These data suggest that F/P repurposing is a promising strategy to improve tissue or organ regeneration.

The first engineered bioartificial pancreas and human bladder [56, 57] have been reported earlier. However, very recently, Takebe et al. were the first to successfully generate functional and well-vascularized human liver bud transplant from pluripotent stem cells as a promising regenerative strategy to address organ donor scarcity [58]. Therefore, it can be expected that biofabrication of other fully biomimetic tissues or organ buds can be achieved in a not so distant future through the integrated application of drug repositioning, stem cell therapy, and tissue engineering that may possibly reduce the demand for organ donors and immune complication. Finally, we believe that researches focused on F/P will continue to grow and that more drugs will be repurposed not only in tissue engineering and regenerative medicine but also in other related fields.

## Figures and Tables

**Table 1:** List of repositioned drugs with their corresponding year of approval, alternative therapeutic uses, and year of repurposing.

Drug name	Year of approval	Original target disease	Year of repositioning	Alternative indication	References
Zidovudine (azidothymidine, AZT)	1964	Cancer	1987	HIV/AIDS	[3]
Amphocin (Amphotericin B)	1955	Fungal infections	1997	Leishmaniasis	[17]
Rogaine (Minoxidil)	1988	Hypertension	1998	Baldness, loss of hair	[14]
Thalidomide (Thalidomide)	1957	Morning sickness	1998 2006	Hansen's disease/multiple myeloma	[18]
Avodart (Dutasteride)	1996	Prostatic hyperplasia	2002	Baldness, loss of hair	[19]
Neurontin (Gabapentin)	2000	Epilepsy	2002	Neuropathic pain	[20]
Symmetrel (Amantadine)	1966	Influenza	2003	Parkinson's disease	[21]
Zyban (Bupropion)	1997	Depression	2005	Smoking cessation	[22]
ReQUIP (Ropinirole)	1998	Parkinson's disease	2005	Restless leg syndrome	[23]
Aspirin (Acetylsalicylic acid)	1899	Inflammation, pain	2006	Antiplatelet aggregation	[13]
Gemzar (Gemcitabine)	1996	Viral disease	2007	Cancer	[24]
Evista (Raloxifene)	1997	Breast cancer	2007	Osteoporosis	[25]
Viagra (Sildenafil)	1998	Angina, hypertension	2007	Erectile dysfunction	[12]
Yentreve (Duloxetine)	2004	Depression	2007	Stress urinary incontinence	[26]
Trexall (Methotrexate)	1946	Cancer	2009	Psoriasis, rheumatoid arthritis	[27]
Parlodel (Bromocriptine)	1988	Parkinson's disease	2009	Diabetes mellitus	[28]

**Table 2:** Applications of F/P micro/nanoparticles (F/P M/NPs) as biomaterials for tissue engineering and regenerative medicine.

Biomaterial uses of F/P micro/nanoparticles (F/P M/NPs)	Reported results	Year of publication	References
(1) Heparin neutralizer	(i) Protamine is a competent heparin neutralizer	1997	[37]
	(ii) Postimplantation administration of protamine reduced the thrombotic complication and remarkably reduced the lengthy bed rest period as well as the hospital stay of the patients		
	(iii) Post-protamine injection reactions of the patients such as transient back pain, hypotension, and skin rashes were well managed in vitro		
	(iv) Severe groin hematoma was observed at a minimal percentage		

(2) Cell carriers	(i) F/P MPs enhanced the viabilities of various stem cells such as hMVECs, human dermal fibroblasts, and ADSCs in suspension culture	2010	[39]
	(ii) F/P MPs adhered into the surfaces of the cells, induced cell aggregation, and promoted cell growth		
	(iii) Cell aggregates secreted increased amount of heparin-binding growth factors such as FGF		
	(iv) F/P MPs induced neovascularization in nude mice model		
	(v) Possible angiogenic biomaterial		
	(i) When F/P MPs were coated on the culture plate, the quiescent state of hepatic stellate RI-T cells (HSCs) was conserved in comparison to those grown under noncoated and matrigel-coated plates	2012	[43]
	(ii) HSCs exhibited suppressed the expressions of collagen I*α*I and TGF-*β* 1 mRNA levels		
	(i) F/P NPs reduced the expansion period of human multilineage ASCs and BMSCs despite the absence of animal serum	2012	[42]
	(ii) F/P NPs induced rapid proliferation rates of ASCs and BMSCs		
	(iii) ASCs and BMSCs maintained their markers and exhibited their rapid multilineage differentiation		
	(iv) F/P NP-coated plates are a useful substratum for the adherence and proliferation of ASCs and BMSCs despite low levels of PRP and FGF-2		
	(i) F/P rapid cell proliferation rate of IR-ASCs under 3D-culture gel system at a low inbred-rat serum content	2013	[40]
	(ii) F/P expedited not only the local cell proliferation but also the vascularization and tissue granulation at the injection sites after transplantation		

(3) Cytokine immobilizer or attractant/biomaterial for cell growth and differentiation	(i) Heparin MPs coating immobilized cytokines, namely, SCF, Tpo, and Flt-3 ligand	2009	[44]
	(ii) Controlled gradual release of the cytokines into the media was demonstrated to occur within 3-4 culture days		
	(iii) Superior CD 34 (+) hematopoietic progenitor cells proliferation rates were shown at approximately 8.0-fold and over 31.9-fold after 6 and after 12 culture days, respectively		
	(i) F/P controlled the gradual release of heparin-binding growth factors like FGF-2 and cytokines such as IL-3 and GM-CSF into the culture media	2009	[41]
	(ii) F/P enhanced the cell growth of hMVECs, human dermal fibroblast cells (hDFCs), and hematopoietic cell line (TF-1) cells when they were used coating agents despite the low level of FBS in the culture media		

(4) Hair growth enhancer	(i) Dalteparin (F; identical to fragmin) and protamine microparticles injection (F/P MPs) facilitated increased hair growth	2011	[7]
	(ii) Microscopic findings showed thickened epithelium, proliferation of collagen fibers and fibroblasts, and increased vessels around follicles		
	(iii) F/P MPs showed a promising therapeutic use in dermatology particularly on hair reconstruction for alopecia		

(5) DNA/protamine injectable dental complex/drug carrier	(i) DNA/protamine complex delayed the growth of certain bacterial species, namely, *Staphylococcusaureus*, *Pseudomonas aeruginosa*, *Porphyromonas gingivalis*, and *Prevotella intermedia *	2011	[6]
	(ii) An effective drug carrier for gum pocket treatment		
	(iii) DNA/protamine complex promoted GTR and GBR		

(6) Protein carrier	(i) F/P MPs are competent carrier of the proteins present in the human PRP that stimulate neovascularization and granulation tissue formation	2011	[46]
	(ii) F/P MPs effectively adsorb growth factors GFs		
	(iii) F/P MPs significantly enhanced neovascularization and filtration of inflammatory cells		
	(i) F/P MPs are good carriers of proteins in PRP and optimized the growth of human aorta endothelial cells (AECs) as well as smooth muscle cells(SMCs)	2012	[45]
	(ii) Superior biological activities of GFs in PRP were demonstrated by the cultured AEC and SMCs treated with F/P MPs		
	(iii) The increases in collateral arteries in ischemic limbs were significantly higher in the PRP-containing F/P MPs group than those in the F/P MPs alone, and PRP alone, in comparison to the control group.		

(7) Injectable biomaterial to prevent skin flap necrosis	(i) Injection of PRP and F/P MPs prior to elevation of skin flaps enriched their survival and prohibited necrosis in rodent models	2011	[8]
	(ii) Histological analysis revealed that the skin flaps preinjected with PRP&F/P MPs exhibited thick granulation of tissues and neovascularization in comparison to the untreated groups		
	(iii) PRP and F/P MPs are a promising injectable biomaterial in reconstructive surgery to prevent skin flap necrosis		

(8) Growth factor (HGF,VEGF) carrier and inducer of neovascularization	(i) FGF-2 was bounded to F/P MPs and facilitated its protections against degradation, controlled release during the culture period	2009	[41]
	(ii) A week after injection, F/P MPs stimulated significant neovascularization and fibrous tissue formation		
	(iii) F/P MPs biodegradation was observed 2 weeks after injection		
	(i) No significant difference in blood pressure among the rabbit animal models of ischemia as revealed by laser Doppler perfusion imaging	2011	[4]
	(ii) A remarkable improvement of blood pressure was observable in animals treated with F/P MPs/FGF-2 compared to the untreated ones		
	(iii) Cotreatment of F/P MPs and FGF 2 significantly induced collateral blood vessel formation in rabbit ischemic models		
	(iv) F/P MPS/FGF 2-induced arteriogenesis and angiogenesis in ischemic limbs present a promising for peripheral artery disease (PAD)		
	(i) HGF-containing F/P MPs substantially enhanced mitogenic effect of HGF on cultured human microvascular endothelial cells	2013	[5]
	(ii) The conjugation of HGF to F/P MPs facilitated the controlled release of HGF and protected these growth factors from heat and proteolytic inactivation		
	(iii) F/P MPs are efficient HGF carriers that facilitate cell proliferation and vascularization of damaged tissues in mice models		
